# Periodontal ligament cells-derived exosomes promote osteoclast differentiation via modulating macrophage polarization

**DOI:** 10.1038/s41598-024-52073-9

**Published:** 2024-01-17

**Authors:** Xinyi Bai, Yingxue Wang, Xinyuan Ma, Yingying Yang, Cong Deng, Mengling Sun, Chen Lin, Linkun Zhang

**Affiliations:** 1 School of Medical, NanKai University, Tianjin, 300071 China; 2 Department of Orthodontics, Tianjin Stomatological Hospital, Tianjin, 300041 China; 3 School of Clinical Stomatology, Tianjin Medical University, Tianjin, 300070 China; 4 Tianjin Key Laboratory of Oral and Maxillofacial Function Reconstruction, 75 Dagu Road, Heping District, Tianjin, 300041 China; 5 Fuzhou Second Hospital, Fuzhou, 350007 China; 6 Tianjin Kanghui Hospital, Tianjin, Tianjin, 300385 China

**Keywords:** Cell biology, Immunology, Medical research

## Abstract

Several studies have demonstrated that exosomes (Exos) are involved in the regulation of macrophage polarization and osteoclast differentiation. However, the characteristics as well as roles of exosomes from human periodontal ligament cells (hPDLCs-Exos) in M1/M2 macrophage polarization and osteoclast differentiation remain unclear. Here, periodontal ligament cells were successfully extracted by method of improved Type-I collagen enzyme digestion. hPDLCs-Exos were extracted by ultracentrifugation. hPDLCs-Exos were identified by transmission electron microscopy (TEM), nanoparticle tracking analysis (NTA) and western blotting (WB). Osteoclast differentiation was evaluated by real-time quantitative polymerase chain reaction (RT-qPCR), WB and tartrate-resistant acid phosphatase (TRAP) staining. M1/M2 macrophage polarization were evaluated by RT-qPCR and WB. The results showed hPDLCs-Exos promoted osteoclast differentiation and M2 macrophage polarization, but inhibited M1 macrophage polarization. Moreover, M1 macrophages inhibited osteoclast differentiation, whereas M2 macrophages promoted osteoclast differentiation. It has shown that hPDLCs-Exos promoted osteoclast differentiation by inhibiting M1 and promoting M2 macrophage polarization.

## Introduction

Orthodontic tooth movement (OTM) occurs in the periodontal steady-state microenvironment through bone remodeling^1^. Periodontal ligament cells (PDLCs) are the most common cells in the periodontal tissues^2,3^. The periodontal ligament is one of fibrous structures that connects the cementum on the tooth root surface to the alveolar bone, aiming anchoring the teeth in the alveolar socket^4^. Exosomes (Exos) are nanovesicles derived from various types of cells. With diameters ranging from 40 to 200 nm^5^, Exos play an important role in intercellular communication^6^. Exos derived from PDLCs, gingival cells and dental pulp cells can play an important role in enhancing the function of recipient cells (proliferation and differentiation, etc.)^7^. PDLCs, which is exposed to OTM for a long time, can perceive mechanical signals and convert them into chemical signals^8,9^. Exosomes from human periodontal ligament cells (hPDLCs-Exos) can be transported to nearby or distant cells, delivering a series of signals that affect the microenvironmental stability of bone remodeling^10^.

OTM is widely recognized as a good reflection of bone immunology because it involves both immune responses and bone remodeling^11^. The interaction between immunity and osteoclasts is of great significance during OTM^1,12^. Macrophages, derived from monocytes^13^, are important members of the immune system^14^ and play crucial roles in tissue repair and inflammation suppression^15^. Macrophages can exhibit different functional phenotypes based on local changes in the microenvironment^16^, and are generally classified into two types: pro-inflammatory M1 macrophages and anti-inflammatory M2 macrophages^17^. Classically activated M1 macrophages are induced by cytokines such as interferon-gamma (IFN-γ) or lipopolysaccharide (LPS), and promote inflammation in the context of innate immunity by producing inflammatory cytokines such as tumor necrosis factor-alpha (TNF-α) and interleukin-6 (IL-6)^18^. In contrast, alternatively activated M2 macrophages are induced by interleukin-4 (IL-4) or interleukin-13 (IL-13), and produce anti-inflammatory cytokines^19^. Osteoclasts, which are bone-resorbing multinucleated cells, also orgin from the monocyte lineage^20^. Macrophage colony-stimulating factor (M-CSF) activation of its receptor c-Fms and receptor activator of the NF-κB ligand (RANKL) activation of receptor activator of the NF-κB (RANK) are important signaling events that prompt osteoclasts (OC) precursors proliferation and differentiation^21^. Although macrophages and osteoclasts share the same precursor cells^22^, the potential impact of macrophages on osteoclastogenesis, particularly the differences between M1 and M2 macrophages, is mainly unknown.

In recent years, Exos have been found in periodontal tissues and have been increasingly studied in relation to orthodontic tooth movement-related bone remodeling^23^. However, there is limited research on the macrophage polarization by hPDLCs-Exos, as well as the potential impact of different types of macrophages on osteoclast differentiation. Here, we hypothesize that hPDLCs-Exos can influence macrophage polarization, thereby participate in osteoclast differentiation.

## Result

### Uptake of hPDLCs-Exos by RAW264.7 macrophages

**Figure 1:**
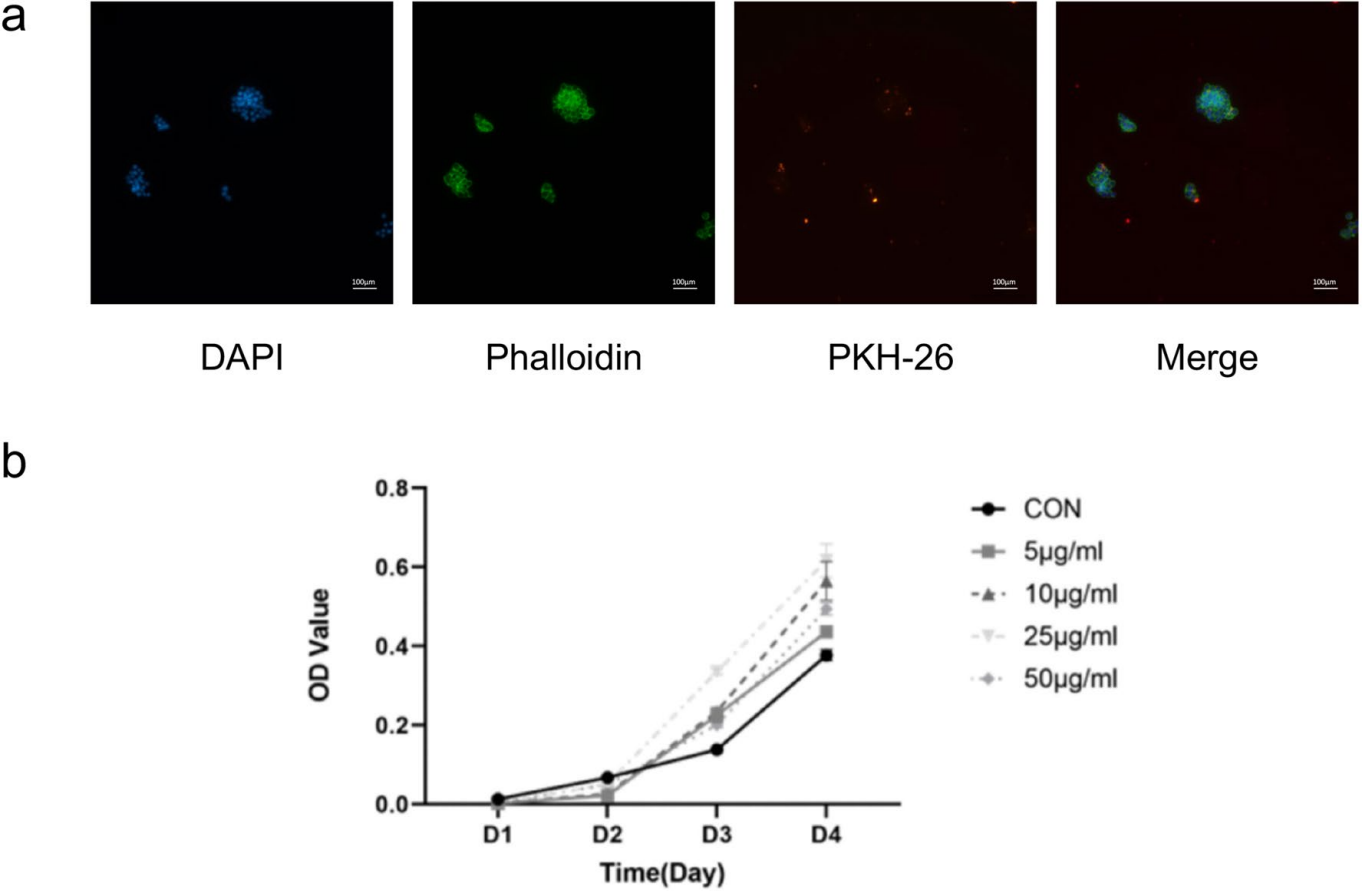
Uptake of hPDLCs-Exos and Osteoclasts. (**a**) RAW264.7 macrophages could uptake hPDLCs-Exos. (scale bar: 100 μm). DAPI labeled the nucleus (blue, round), Phalloidin labeled RAW264.7 macrophages (green, circular), and PKH-26 labeled exosomes (red, punctiform). (**b**) CCK-8. Osteoclasts were induced by M-CSF and RANKL for 6 days. (**c**) TRAP staining. (scale bar: 100 μm). (**d**) RT-qPCR showed mRNA expression levels of osteoclasts marker genes: *Acp5*, *Mmp9* and *Ctsk.* (*P < 0.05, **P < 0.01, ***P < 0.001, ****P < 0.0001).

The isolation and identification of hPDLCs-Exos were conducted following the protocols described in previous article^24^. Ultracentrifugation was employed for exosome isolation, while TEM, NTA and WB were utilized for exosome characterization. Confocal microscopy showed that hPDLCs-Exos labeled by PKH-26 were taken up by RAW264.7 macrophages (Fig. 1a). The CCK-8 results demonstrated that exosomes of different concentrations exhibited the promoting effect on cell proliferation. Meanwhile the most significant promotion was observed at a concentration of 25 µg/ml. The differences were found to be statistically significant (Fig. 1b). Therefore, 25 µg/ml exosomes were selected for induction in subsequent experiments.

### hPDLCs-Exos promote osteoclast differentiation of RAW264.7 macrophages

**Figure 2:**
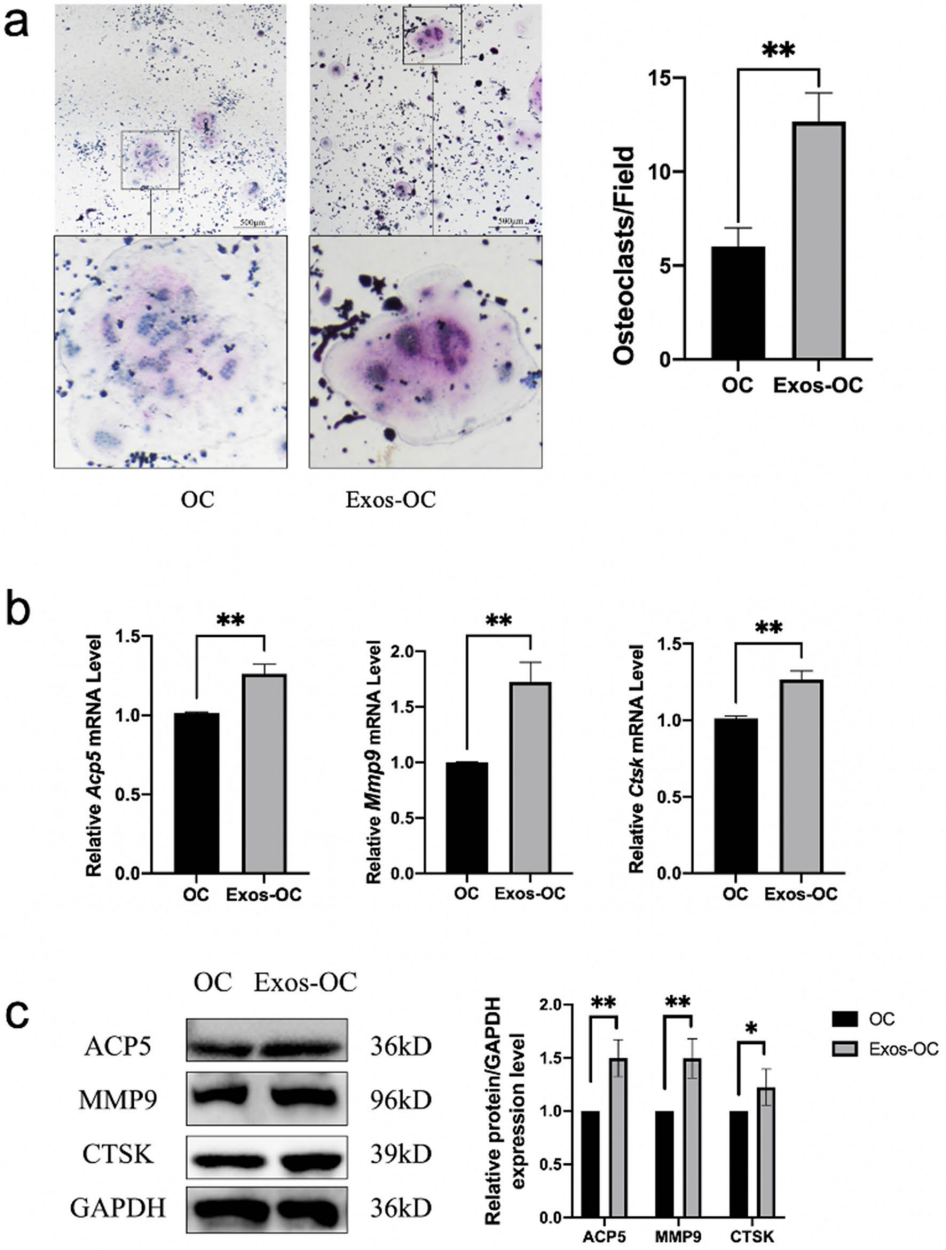
hPDLCs-Exos promote osteoclast differentiation. OC group was treated with exosome-free α-MEM medium for 1 day and M-CSF and RANKL for 6 days, while the Exos-OC group was treated with exosomes for 1 day followed by M-CSF and RANKL for 6 days. (**a**) TRAP staining. (scale bar: 500 μm). (**b**) RT-qPCR showed mRNA expression levels of osteoclasts marker genes: *Acp5*, *Mmp9* and *Ctsk*. (**c**) WB analysis was used to detect osteoclasts marker proteins: ACP5, MMP9 and CTSK. (**P < 0.01, ***P < 0.001).

Osteoclasts were induced successfully using M-CSF and RANKL (Appendix Fig. 1). RAW264.7 macrophages treated with hPDLCs-Exos (Exos-OC group) had a significantly higher osteoclast differentiation potential than OC group, as demonstrated by TRAP staining (Fig. 2a). The RT-qPCR results showed a statistically significant increase in the expression of osteoclast marker genes *Acp5*, *Mmp9*, and *Ctsk* in Exos-OC group, compared with OC group (Fig. 2b). The expression of osteoclast marker proteins was significantly higher in Exos-OC group compared to OC group, as demonstrated by WB analysis. The difference was statistically significant (Fig. 2c). These results indicated that hPDLCs-Exos enhance osteoclast differentiation on RAW264.7 macrophages, compared with the control group.

### hPDLCs-Exos promote osteoclast M2 macrophage polarization, but inhibited M1 macrophage polarization

**Figure 3:**
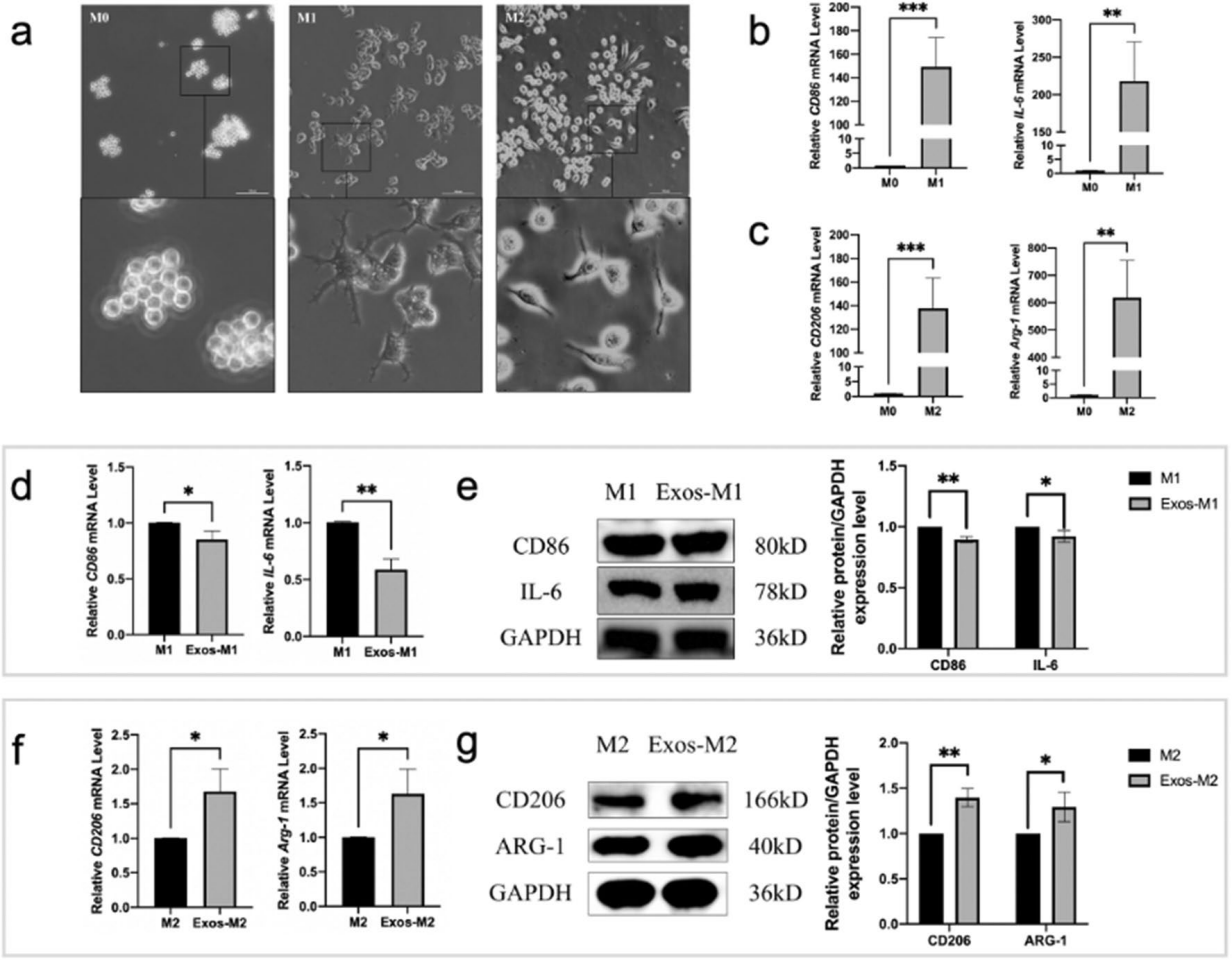
hPDLCs-Exos inhibit M1 macrophage polarization and promote M2 macrophage polarization. (**A**) Morphology of M0, M1 and M2 macrophages. (Scale bar: 100 μm). (**B**) The mRNA expression levels of M1 macrophages marker genes were detected by RT-qPCR. (**C**) The mRNA expression levels of M2 macrophages marker genes were detected by RT-qPCR. M1 group was treated with exosome-free α-MEM culture medium for 1 day, followed by LPS and IFN-γ for 1 day; Exos-M1 group was treated with hPDLCs-Exos for 1 day, followed by LPS and IFN-γ for 1 day. M2 group was treated with exosome-free α-MEM culture medium for 1 day, followed by IL-4 for 2 days, Exos-M2 group was treated with hPDLCs-Exos for 1 day, followed by IL-4 for 2 days. (**D**) RT-qPCR showed mRNA expression levels of M1 macrophages marker genes. (**E**) RT-qPCR showed mRNA expression levels of M2 macrophages marker genes. (**F**) WB analysis detected the expression levels of M1 macrophages marker proteins. (**G**) WB showed the expression levels of M2 macrophages marker proteins. (*P < 0.05, **P < 0.01, ***P < 0.001, ****P < 0.0001).

Firstly, M1 macrophages were induced by LPS and IFN-γ, and M2 macrophages were induced by IL-4. Under Ti-S inverted microscope (Nikon, Japan), it was observed that M0 macrophages (RAW264.7 macrophages without induction) were circular, M1 macrophages had multiple protrusions, and M2 macrophages were spindle-shaped with two protrusions (Fig. 3a). The mRNA expression levels of the *CD86* and *IL-6* genes in M1 group, which are markers of M1 macrophages, were significantly increased with a statistically significant difference, compared with M0 group (Fig. 3b). Furthermore, the mRNA expression levels of *CD206* and *Arg-1* in M2 group, which are markers of M2 macrophages, were significantly increased with a statistically significant difference, compared with M0 group (Fig. 3c).

Treatment of RAW264.7 macrophages with hPDLCs-Exos (Exos-M1 group) showed that the mRNA expression levels of *CD86* and *IL-6* were significantly reduced (Fig. 3d), while the mRNA expression levels of *CD206* and *Arg-1* in Exos-M2 group were significantly increased compared to M1 or M2 group (Fig. 3f). WB results showed that, compared with M1 group, the protein expression levels of CD86 and IL-6 significantly decreased in Exos-M1 group (Fig. 3e). Compared to M2 group, the protein expression levels of CD206 and ARG-1 increased significantly in Exos-M2 group (Fig. 3g).

### M2 macrophages are more effectively differentiated into osteoclasts than M1 macrophages

**Figure 4:**
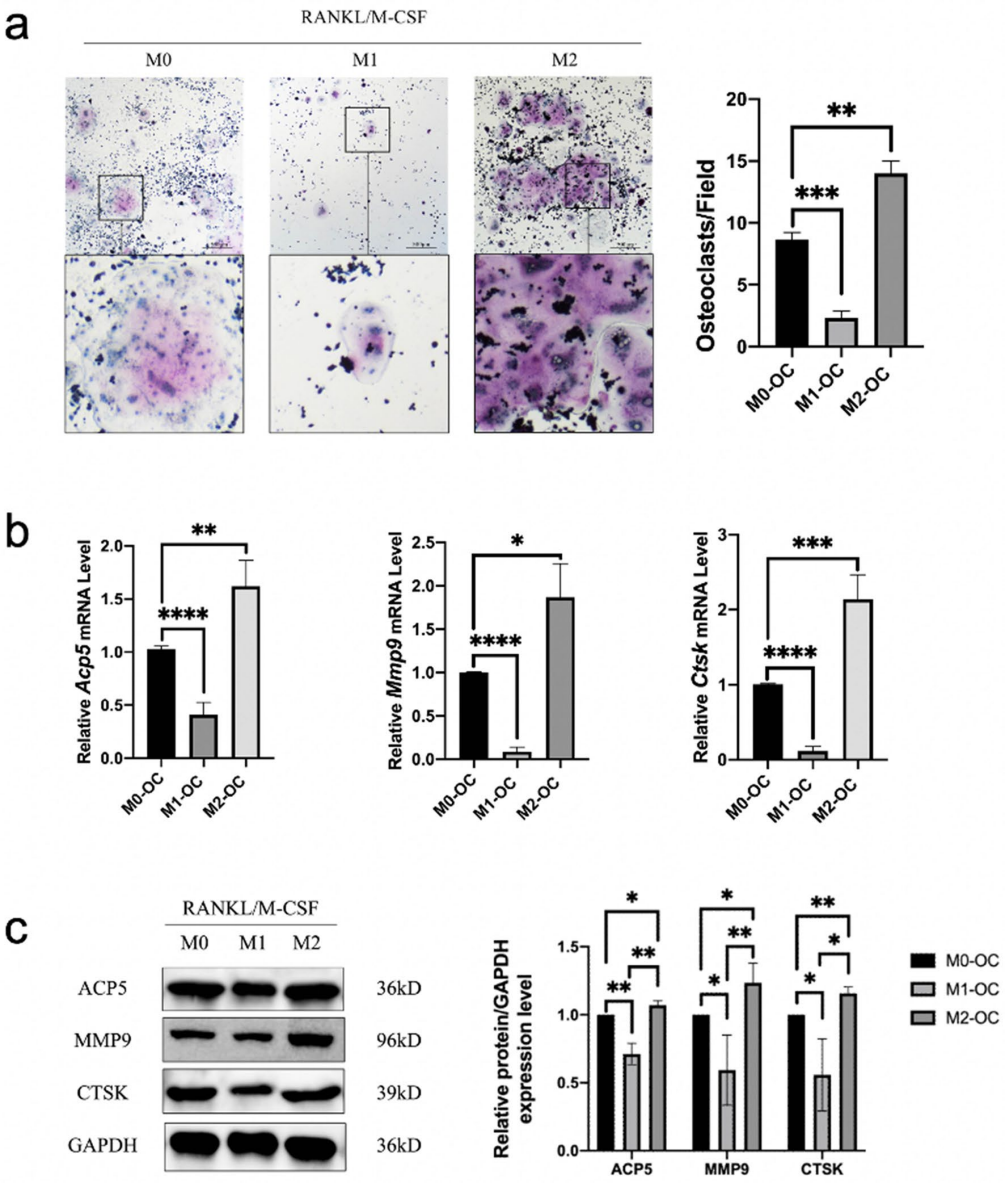
Compared to M1, M2 macrophages is more prone to differentiate into osteoclasts. M0-OC group was treated with exosome-free α-MEM medium for 2 days, followed by M-CSF and RANKL for 6 days. M1-OC group was treated with exosome-free α-MEM medium for 1 day, LPS and IFN-γ for 1 day, followed by M-CSF and RANKL for 6 days. M2-OC group was treated with IL-4 for 2 days, followed by M-CSF and RANKL for 6 days. All the aforementioned groups were sampled on the 8th day. (**a**) TRAP staining. (scale bar: 500 μm). (**b**) RT-qPCR showed mRNA expression levels of osteoclasts marker genes: *Acp5*, *Mmp9* and *Ctsk*. (**c**) Western blot showed the expression levels of osteoclasts marker proteins: ACP5, MMP9, and CTSK. (*P < 0.05, **P < 0.01, ***P < 0.001, ****P < 0.0001).

To determine the effect of M1 and M2 macrophages on osteoclast differentiation, RAW264.7 macrophages were first treated with LPS and IFN-γ or IL-4, followed by stimulation with M-CSF and RANKL. Compared with M0-OC group, osteoclast differentiation in M1-OC group was significantly reduced. On the contrary, osteoclast differentiation was significantly increased in M2-OC group, with a statistically significant difference (Fig. 4a). Compared to M0-OC group, the mRNA expression levels of osteoclasts marker genes *Acp5*, *Mmp9*, and *Ctsk* significantly decreased in M1-OC group, while significantly increased in M2-OC group (Fig. 4b). Similarly, there were comparable expression outcomes at the protein level (Fig. 4c).

## Discussion

The periodontal microenvironment plays a crucial role in OTM and bone remodeling^25^. Both PDLCs and osteoclasts are key cells in periodontal tissue. Exosomes internalized by periodontal ligament stem cells have an impact on bone metabolism via releasing the contents effectively^26^. Previous research has shown that periodontal ligament fibroblasts-derived exosomes induced by Prostaglandin E_2_ inhibit human periodontal ligament stem cells osteogenic differentiation^24^. In the pressure zone of OTM in rats, the expression of Interleukin-17 (IL-17) can be detected. Simultaneously, by adding exogenous recombinant IL-17, the expression of RANKL is upregulated, promoting osteoclast differentiation^27^. However, the role of hPDLCs-Exos in osteoclast differentiation and bone immunology remains unclear.

In this study, we validated the impact of hPDLCs-Exos on osteoclast differentiation and macrophage polarization, as well as the relationship between them. Under the stimulation of hPDLCs-Exos, Acp5, Mmp9 and Ctsk (OC-specific markers^28^), along with CD206 and Arg-1 (M2 macrophage-specific markers^29^) were highly expressed. Conversely, CD86 and IL6 (M1 macrophage-specific markers^29,30^) showed low expression, compared with control group. Compared to M0 group, osteoclast-specific markers showed low expression in M1 group, while high expression was observed in M2 group. To sum up, hPDLCs-Exos promote osteoclast differentiation by inhibiting M1 macrophage polarization and boosting M2 macrophage polarization. Exos contain bioactive substances, including proteins, RNAs, lipids and cytokine receptors^31^, and are present in various body fluids^32^. Exos are involved in intercellular signaling and regulation, making them a hot research topic in biomedical fields^33^. Previous studies have shown that Exos play a crucial role in bone immunology and remodeling^33,34^. Although extensive research has been conducted on the effect of Exos on bone remodeling^35^, studies on bone immunology and osteoclast differentiation remain limited. Huang Huaming et al. demonstrated that mechanical force promotes osteoclast differentiation through the Exos protein ANXA3 secreted by periodontal ligament stem cells^36^, which is similar to the results of this study showing that hPDLCs-Exos promote osteoclast differentiation. Notably, the results of this study also suggest that the effect of hPDLCs-Exos on osteoclast differentiation is partially dependent on changes in M1 and M2 macrophage polarization. Yuki Nakao et al. reported that Exos from TNF-α-treated human gingiva-derived MSCs enhance M2 macrophage polarization^17^. Wang et al. argued that GMSC-derived exosomes may promote M1 macrophage transformation into M2 macrophages. Similarly, this study shows that hPDLCs-Exos can inhibit M1 macrophage polarization and promote M2 macrophage polarization.

Although the impact of immune responses on bone metabolism is widely acknowledged in bone immunology studies^37^, the possible influence of macrophage subtypes on osteoclast differentiation has been controversial. M1 macrophages are known to secrete a variety of pro-inflammatory cytokines that facilitate osteoclast differentiation; conversely, M2 macrophages produce anti-inflammatory cytokines that suppress the formation of osteoclasts. Nonetheless, transcriptional regulation within different macrophage subtypes significantly affects their ability to become osteoclasts. Specifically, M1-related transcription factors such as IRF5 and IRF8 strongly impede osteoclast formation, whereas the M2-related transcription factor IRF4 substantially boosts this process^38^. Yang et al. demonstrated that, compared to M1 macrophages, M2 macrophages exhibit elevated expression of osteoclast differentiation markers, presenting more pronounced sealing zones, and notably larger resorption areas on calcium phosphate-coated plates^39^. Studies indicate that osteoclasts express certain traits associated with M2 macrophages, such as CD163, CD206 and IL-10, suggesting a possible origin from M2 macrophage fusion^40,41^. Through the secretion of IFN-γ and IL-12, M1 macrophages increase osteoclast apoptosis, thereby inhibiting osteoclast differentiation, and can mitigate alveolar bone loss in ligature-induced periodontitis by transitioning to M1 macrophages^18^. Furthermore, rosiglitazone significantly promotes M2 polarization under the influence of osteoclast inducers, thereby enhancing osteoclast differentiation^40^, further demonstrating the greater osteoclastogenic potential of M2 macrophages. This study, utilizing the RAW264.7 macrophage line, revealed that M1 macrophages inhibit while M2 macrophages promote osteoclast differentiation, aligning with the findings of Tsuguno Yamaguchi^18^ and Yang^40^.

Dynamic changes in macrophage subtypes are reportedly linked to inflammation and disease resolution. M2 macrophages treated with LPS can transform into M1 macrophages^42^. When M1 macrophages are transfected with IL-4 and IL-10 expression vectors, they can be re-polarized into M2 macrophages^43^. Early stages of bacterial and viral infections prompt macrophage polarization towards M1, while chronic inflammation aids in the transition from M1 to M2^44^. Hence, diseases or interventions that induce chronic inflammation and cytokine expression changes may influence macrophage subtype transitions, thereby affecting osteoclast differentiation capabilities. In the periodontal environment during OTM, monocytes/macrophages from systemic circulation infiltrate periodontal tissues and secrete cytokines, exhibiting a typical sterile inflammatory response that is acute at first but becomes chronic with the activation of orthodontic appliances, accompanied by the proliferation of fibroblasts and osteoclasts^45^.

This study demonstrated that hPDLCs-Exos promote osteoclast differentiation, which is attributed to the influence of M1 and M2 macrophage polarization. Moreover, these novel findings regarding the ability of hPDLCs-Exos to inhibit M1 and promote M2 macrophage polarization could facilitate the development of therapeutic strategies for diseases associated with M1 or M2 macrophages. In this study, we did not analyze the contents of hPDLCs-Exos. Therefore, the mechanisms by which exosomes regulate macrophage polarization and osteoclast differentiation remain to be further studied, which might be due to microRNA, mRNA, and proteins contained within^46^. Additionally, the specific mechanisms by which macrophage subtypes regulate osteoclast differentiation and the influence of macrophages from different sources on osteoclast differentiation require further exploration. Our team will also continue to investigate whether transcription factors from different macrophage subtypes affect the osteoclastogenic potential. There are many factors that may affect the release of Exos from PDLCs, such as mechanical^36^, inflammatory^24^, and oxidative stress^47^ conditions. It's crucial to explore methods that can accelerate the secretion of Exos from PDLCs. This pursuit will assist in harnessing hPDLCs-Exos to enhance osteoclast differentiation.

In conclusion, these findings may have significant implications for reconstructing the favorable immune microenvironment in periodontal tissues for osteoclast differentiation. Futhermore, they may also provide insights to accelerate OTM.

## Material and methods

### Cell culture

The acquisition and cultivation of PDLCs, as well as the isolation and identification of hPDLCs-Exos, were conducted following the protocols described in previous article^24^. RAW264.7 (CL-0190) macrophages were kindly provided by Procell Life Science& Technology Co., Ltd. The cells were cultured in DMEM (Gibco, Invitrogen, USA) with 10% fetal bovine serum (FBS) (Tianhang, China) and 1% penicillin/streptomycin (Beyotime, China) at 37 °C with 5% CO_2_ in a humidified incubator. RAW264.7 macrophages were loosely adherent and passaged every three days.

### Labeling and uptake of Exosomes

Exosomes were labeled with PKH-26 (Sigma-Aldrich, USA) according to the instructions. The PKH-26-labeled exosomes were then co-incubated with RAW264.7 macrophages for 24 h, fixed with 4% paraformaldehyde, and stained with DAPI (Beyotime, China) for 1 min. Finally, the uptake of PKH26-Exo in RAW264.7 macrophages was observed under an inverted fluorescence microscope (Nikon, Japan).

### Reagents and cell treatment

RAW264.7 macrophages were seeded at a density of 1.5 × 10^4^ cells per well in a 6-well plate and cultured for 24 h. After cells adhered to the wall, they were washed once with PBS and induced as follows: (a) Osteoclast differentiation: cells were induced with 100 ng/mL RANKL (Sino Biological, China) and 30 ng/mL M-CSF (Sino Biological, China) for 6 days with medium changed every 2 days; (b) M1 macrophage polarization: cells were induced with 100 ng/mL LPS (Solarbio Science & Technology, China) and 20 ng/mL IFN-γ (Sino Biological, China) for 24 h; (c) M2 macrophage polarization: cells were induced with 20 ng/mL IL-4 (ACRO Biosystems, USA) for 48 h. All treatments were performed in complete medium containing 10% exosome-free FBS. To obtain exosome-free FBS, FBS was ultracentrifuged at 130, 000×*g* for 4 h, at 4 °C in advance.

### CCK-8 assay

RAW264.7 macrophages were seeded at a density of 10^3^ cells per well in a 96-well plate and cultured in exosome-free medium for 24 h. After cells adherence, they were washed once with PBS and induced with different concentrations of exosomes for 24 h: 0 µg/ml, 5 µg/ml, 10 µg/ml, 25 µg/ml, 50 µg/ml. At 1, 2, 3 and 4 days after the intervention, a group of 96-well plates were taken and washed with PBS, 10 µl of CCK-8 reagent (Solarbio, China) was added to each well. The plates were then incubated in the dark at 37 °C for 1 h. Thereafter, the optical density (OD) obtained from the microplate reader (Tecan, Switzerland) at 450 nm was collected.

### Real-time quantitative PCR (RT-qPCR) assay

Total RNA was extracted by Takara kit (Takara, Japan) on ice. Concentration and purity were measured by the NanoDrop One Microvolume UV–Vis Spectrophotometer (Kaiao, China). The cDNA was synthesized using the Prime Script RT Reagent Kit (Takara, Japan). SYBR Premix Ex Taq II kit (Takara, Japan) was used for real-time PCR analysis by the Roche Light Cycler 480 sequence detection system (Roche Diagnostics, Switzerland). GAPDH was used as the reference gene. The primer sequences for the relevant genes are shown in Appendix Table 1.

### Western blotting (WB) and antibody

Total protein was extracted by lysing cells on ice for 30 min with RIPA buffer (Beyotime, China). Protein concentration was measured using the BCA Protein Assay Kit (Beyotime, China). Equal amounts of protein were separated by SDS-PAGE gel electrophoresis and transferred onto the polyvinylidene difluoride (PVDF) membrane. After being blocked at room temperature for 1 h, the PVDF membrane was incubated overnight with primary antibody at 4 °C. After being washed with TBST and TBS, the PVDF membrane was incubated with secondary antibody at room temperature for 1 h. Finally, the protein bands were visualized using an ECL chemiluminescence kit (Millipore, USA).

The primary antibodies were listed in the following: ACP5 (ABclonal, #A16338, 1:1000, USA), MMP9 (ABclonal, #A0289, 1:1000, USA), CTSK (ABclonal, #A5871, 1:1000, USA), CD86 (ABclonal, #A19026, 1:1000, USA), IL-6 (ABclonal, #A1570, 1:1000, USA), CD206 (ABclonal, #A11192, 1:1000, USA), ARG-1 (ABclonal, #A1847, 1:1000, USA), GAPDH (Beyotime, #AF5009, 1:1000, China).

### Tartrate-resistant acid phosphatase (TRAP) staining

The acid phosphatase assay kit (Sigma-Aldrich) is used for detection, and the specific steps are listed as follows: cells were washed twice with PBS, fixed by 4% paraformaldehyde for 5–10 min. Afterwards, cells are incubated with TRAP substrate solution at room temperature for 60 min, and then stained with hematoxylin for 50 s. Osteoclasts are identified as TRAP-positive multinucleated cells containing more than three nuclei.

### Statistical analysis

Quantitative data were presented as mean ± standard deviation (SD) and experiments were independently repeated at least three times. Independent sample *t*-test and one-way ANOVA were used for statistical analysis, with a significance threshold set at *P* < 0.05. Statistical analysis was performed using SPSS 17.0 (IBM, USA).

## Conclusion

**Figure 5:**
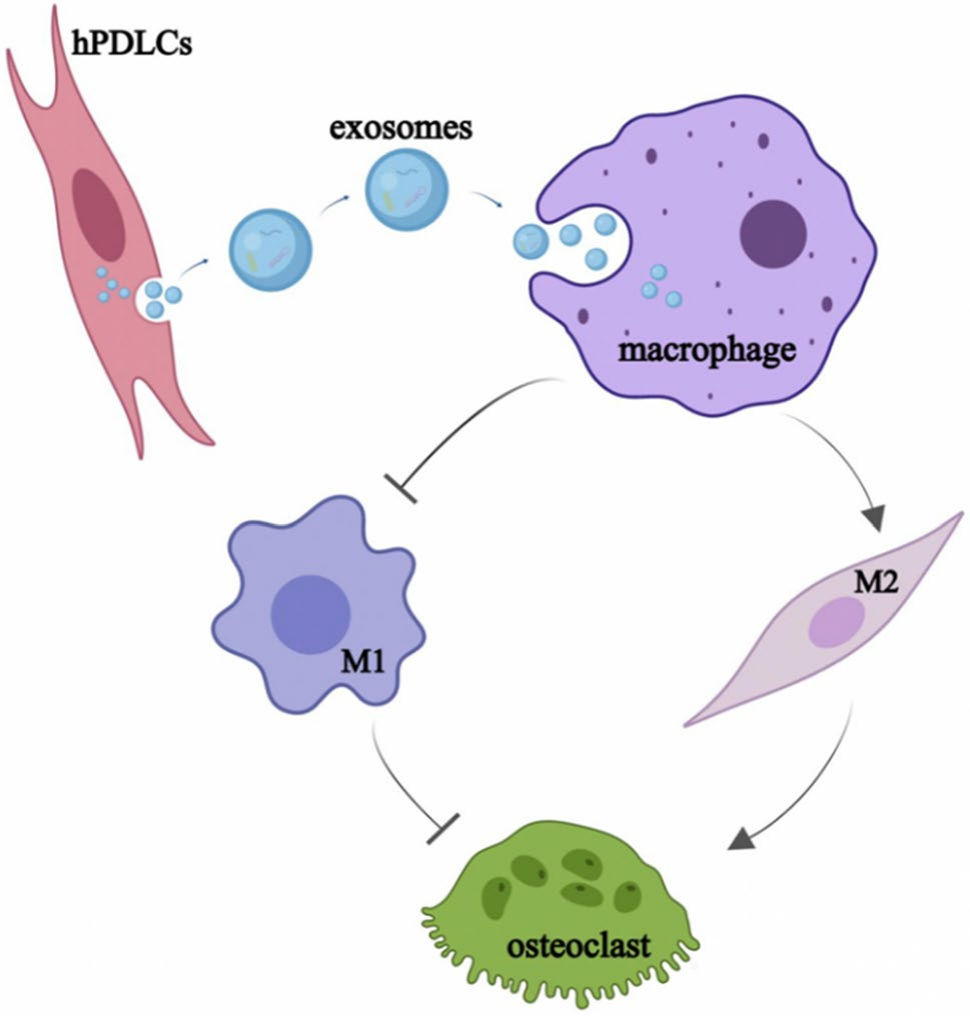
Experimental research mechanism diagram. hPDLCs-Exos can suppress M1 macrophage polarization and promote M2 macrophage polarization, thereby promoting osteoclast differentiation.

Exosomes derived from human periodontal ligament cells can suppress M1 macrophage polarization and promote M2 macrophage polarization, thereby promoting osteoclast differentiation (Fig. 5).

### Supplementary Information

Supplementary Information.

## Data Availability

The datasets used and/or analysed during the current study available from the corresponding author on reasonable request.
