# Author Correction: Periodontal ligament cells-derived exosomes promote osteoclast differentiation via modulating macrophage polarization

**DOI:** 10.1038/s41598-024-53412-6

**Published:** 2024-02-07

**Authors:** Xinyi Bai, Yingxue Wang, Xinyuan Ma, Yingying Yang, Cong Deng, Mengling Sun, Chen Lin, Linkun Zhang

**Affiliations:** 1 School of Medical, NanKai University, Tianjin, 300071 China; 2 Department of Orthodontics, Tianjin Stomatological Hospital, Tianjin, 300041 China; 3 School of Clinical Stomatology, Tianjin Medical University, Tianjin, 300070 China; 4 Tianjin Key Laboratory of Oral and Maxillofacial Function Reconstruction, 75 Dagu Road, Heping District, Tianjin, 300041 China; 5 Fuzhou Second Hospital, Fuzhou, 350007 China; 6 Tianjin Kanghui Hospital, Tianjin, 300385 China

Correction to: *Scientific Reports* 10.1038/s41598-024-52073-9, published online 17 January 2024

In the original version of this Article Xinyi Bai and Yingxue Wang were omitted as equally contributing authors.

The original Article has been corrected.

