# Supplementary material for: Periodontal ligament cells-derived exosomes promote osteoclast differentiation via modulating macrophage polarization
Source: Sci Rep. 2024 Jan 17;14:1465. doi: 10.1038/s41598-024-52073-9 (PMC10794214; doi:10.1038/s41598-024-52073-9)
Supplement: Supplementary file 1 — Supplementary Information. [file 41598_2024_52073_MOESM1_ESM.pdf]

Periodontal ligament cells-derived exosomes promote osteoclast differentiation via modulating macrophage polarization

Appendix Table 1 primer sequences

Genes	Forward primer (5’-3’)	Reverse primer (5’-3’)
Acp5	GATGCCAGCGACAAGAGGTT	CATACCAGGGGATGTTGCGAA
Mmp9	GCAGAGGCATACTTGTACCG	TGATGTTATGATGGTCCCACTTG
Ctsk	CAGCAGAACGGAGGCATTGA	CTTTGCCGTGGCGTTATACATACA
CD86	ACGGAGTCAATGAAGATTTCCT	GATTCGGCTTCTTGTGACATAC
IL6	CTTGGGACTGATGCTGGTGAC	TTCTCATTTCCACGATTTCCCA
CD206	ACCTGGCAAGTATCCACAGCATTG	TGTTGTTCTCATGGCTTGGCTCTC
Arg1	ATGTCCCTAATGACAGCTCCT	GCTTCCAACTGCCAGACTGT
Gapdh	GAGAGTGTTTCCTCGTCCCG	ACTGTGCCGTTGAATTTGCC

Appendix Figure 1

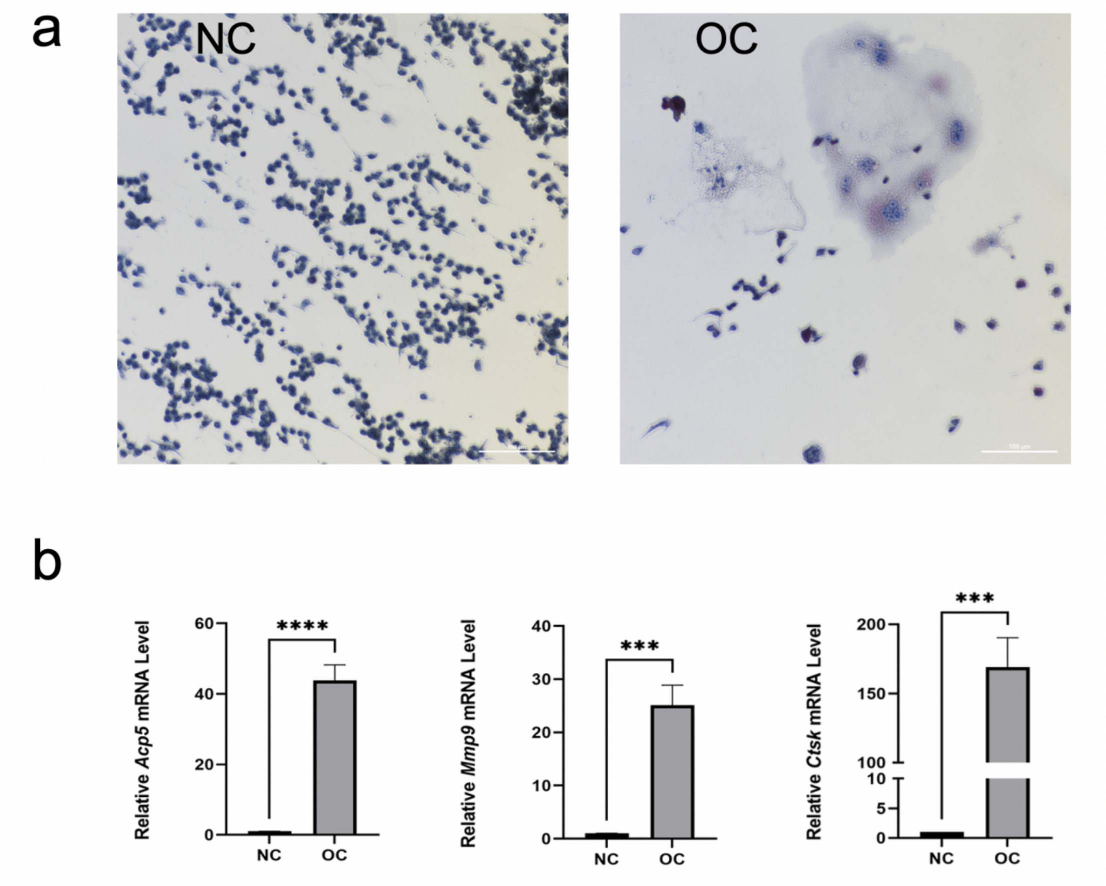

Osteoclasts were induced by M-CSF and RANKL for 6 days. (C) TRAP staining (scale bar: 100 μm). (d) RT-qPCR showed mRNA expression levels of osteoclasts marker genes: *Acp5*, *Mmp9* and *Ctsk*. ($^{***}P$<0.001, $^{****}P$<0.0001)

EXO&OC

ACP5

	ACP5	GAP
Sample 1	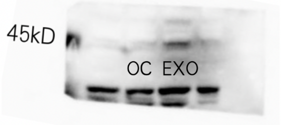	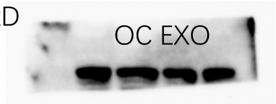
Sample 2	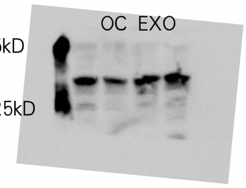	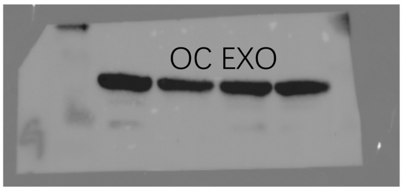
Sample 3	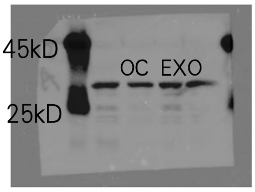	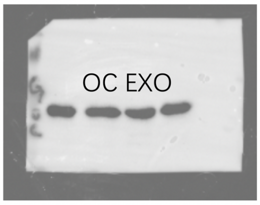

MMP9

	MMP9	GAP
Sample 1	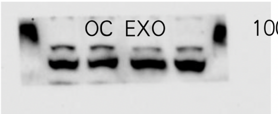	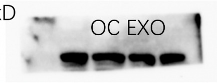
Sample 2	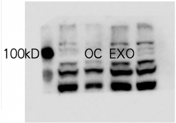 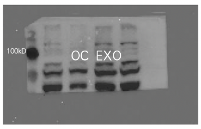 These two blots are the same WB images, and they are all placed here to see the edges of bolt.	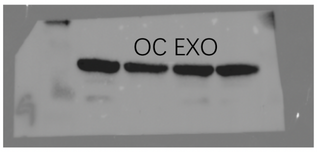
Sample 3	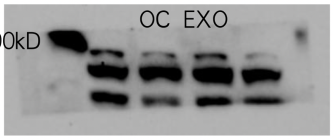	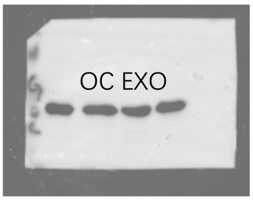

CTSK

	CTSK	GAP
Sample 1	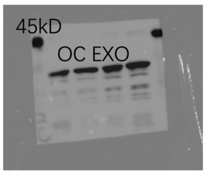	45kD 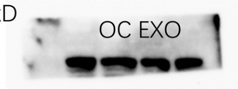
Sample 2	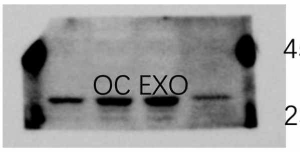 45kD 25kD	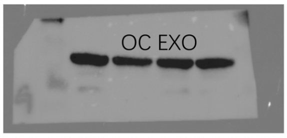
Sample 3	45kD 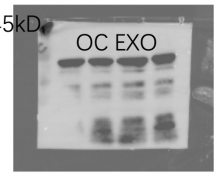	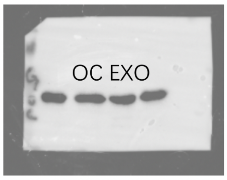
Sample 4	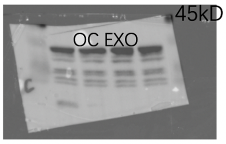 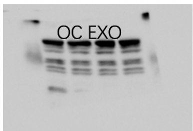 These two blots are the same WB images, and they are all placed here to see the edges of bolt.	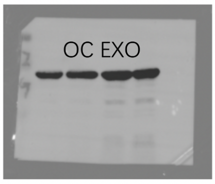

EXO&M1

CD86

	CD86	GAP
Sample 1	100kD 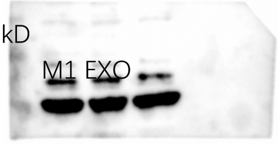	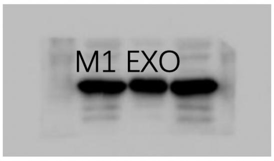
Sample 2	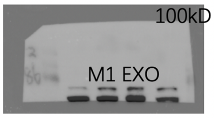 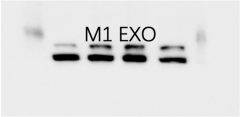 These two blots are the same WB images, and they are all placed here to see the edges of bolt.	45kD 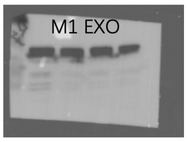
Sample 3	100kD 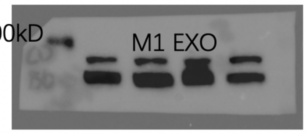	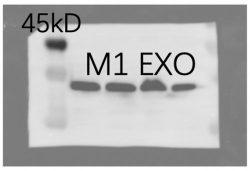

IL-6

	IL-6	GAP
Sample 2	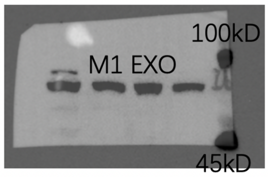	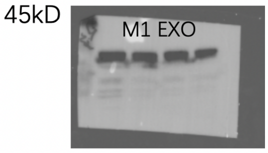
Sample 3	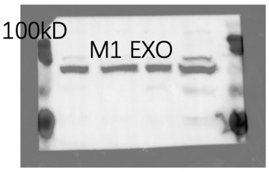	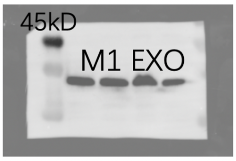
Sample 4	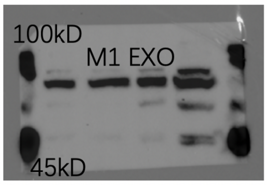	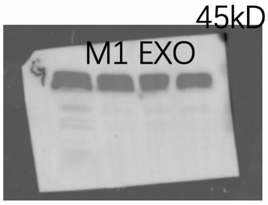

<u>EXO&M2</u>

CD206

	CD206	GAP
Sample 1	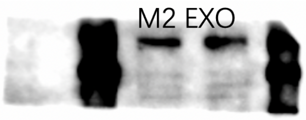	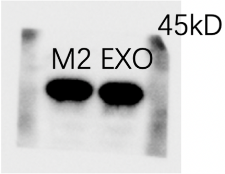
Sample 2	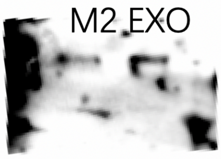	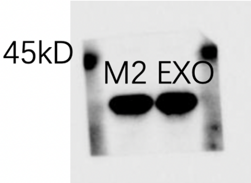
Sample 3	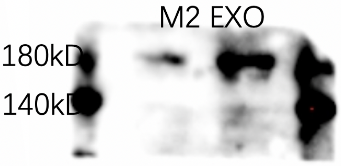	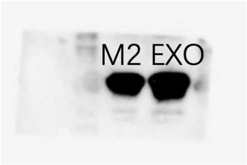

ARG-1

	ARG-1	GAP
Sample 1	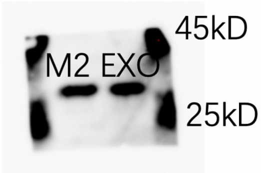	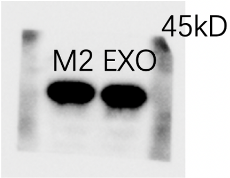
Sample 2	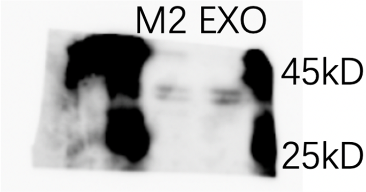	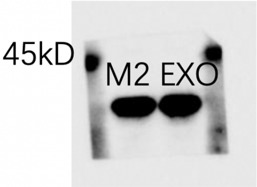
Sample 3	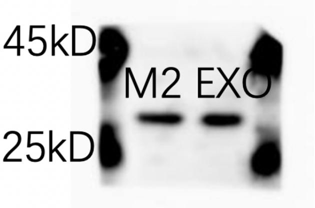	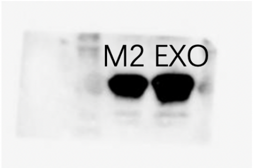

M1/M2&OC

ACP5

	ACP5	GAP
Sample 1	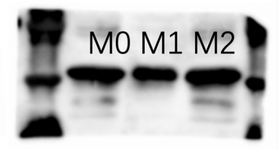	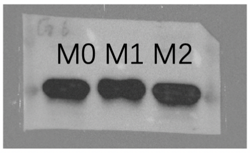
Sample 2	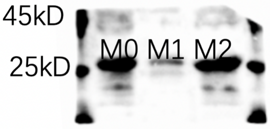	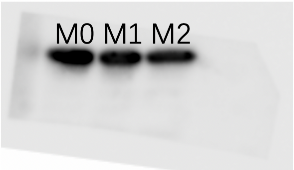
Sample 3	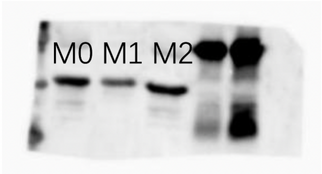	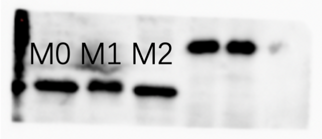

MMP9

	MMP9	GAP
Sample 4	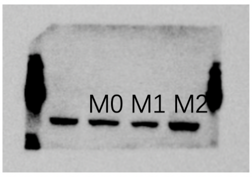	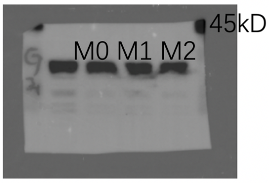
Sample 5	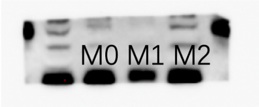	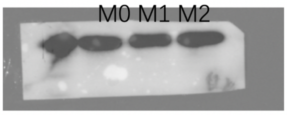
Sample 6	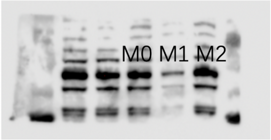	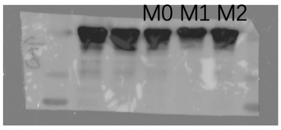
Sample 7	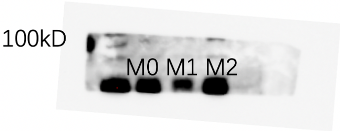	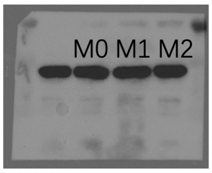

CTSK

	CTSK	GAP
Sample 1	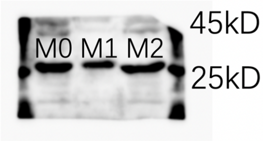	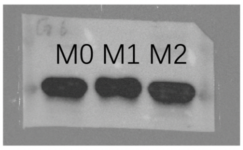
Sample 4	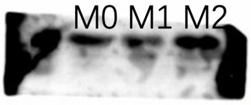	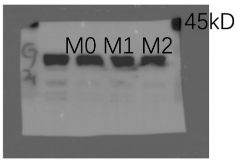
Sample 5	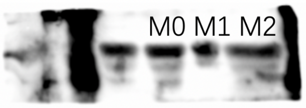	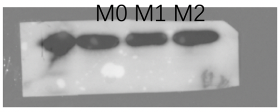
Sample 7	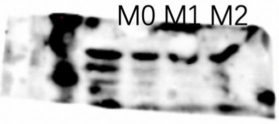	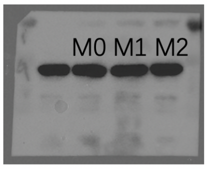

Author

Co-first author:

Xinyi Bai, 2120211558@mail.nankai.edu.cn, 1. Nankai University, school of medical; 2. Tianjin Stomatological Hospital, Department of Orthodontics

Yingxue Wang, 15900370885@163.com, 1. Tianjin Kanghui Hospital

Other authors:

Xinyuan Ma, 634424830@163.com, 1. Tianjin Stomatological Hospital, Department of Orthodontics; 2. Tianjin Medical University, School of Clinical Stomatology

Yingying Yang, 787581660@qq.com, 1. Fuzhou Second Hospital

Cong Deng, 1450104341@qq.com, 1. Nankai University, school of medical; 2. Tianjin Stomatological Hospital, Department of Orthodontics

Mengling Sun, 1104861126@qq.com, 1. Tianjin Stomatological Hospital, Department of Orthodontics; 2. Tianjin Medical University, School of Clinical Stomatology

Corresponding author:

Chen Lin, linchen@tmu.edu.cn, 1. Tianjin Stomatological Hospital, Department of Orthodontics; 2. Tianjin Stomatological Hospital, Tianjin Key Laboratory of Oral and Maxillofacial Function Reconstruction

Linkun Zhang, linkunzhang@nankai.edu.cn, 1. Tianjin Stomatological Hospital, Department of Orthodontics; 2. Tianjin Stomatological Hospital, Tianjin Key Laboratory of Oral and Maxillofacial Function Reconstruction
